# Application of Machine Learning Algorithms for Evaluating Predictors and Developing Diagnostic Models for Female Infertility Classification

**DOI:** 10.3390/bioengineering13070782

**Published:** 2026-07-07

**Authors:** Anwesha Dey, Sandipan Das, Rinku Saha, Filomena Mottola, Kushal Kumar Kar, Yogisharadhya Revanaiah, Israel Maldonado Rosas, Shubhadeep Roychoudhury

**Affiliations:** 1 Department of Life Science and Bioinformatics, Assam University, Silchar 788011, India; 2 Mediland Hospital and Research Centre, Silchar 788001, India; 3 Department of Environmental, Biological and Pharmaceutical Sciences and Technologies, University of Campania Luigi Vanvitelli, 81100 Caserta, Italy; 4 ICAR-National Institute of High Security Animal Diseases, Bhopal 462022, India; 5 Citmer Reproductive Medicine, Mexico City 11520, Mexico

**Keywords:** women’s health, fertility, predictive models, anti-Müllerian hormone, PMOS, PCOS

## Abstract

Infertility affects millions worldwide, with estimates indicating that 1 in 6 people of reproductive age will experience it in their lifetime. Globally, infertility impacts between 12.6–17.5% of couples of reproductive age. Recently, machine learning (ML) has garnered significant attention in biomedical research, enabling creation of predictive models that can personalize disease treatment based on measurable variables, thereby aiding in the development of diagnostic tools. In this study, 28 predictor variables were selected preliminarily; after a multicollinearity test, 20 predictors were selected for the classification task and modelled as a binary supervised classification problem. Seven ML algorithms were evaluated, including Logistic Regression, Random Forest, Decision Tree, Support Vector Machine, Naïve Bayes, K-Nearest Neighbour, and Extreme Gradient Boosting (XGBoost). Statistical analysis showed that anti-Müllerian hormone (AMH) can serve as a biomarker for diagnosing PCOS and evaluating ovarian reserve. Female fertility has been associated negatively with waist circumference (r = −0.35), systolic blood pressure (r = −0.30), poor ovarian reserve (r = −0.28), and triglycerides (r = −0.33), suggesting a possible link between these metabolic factors and female infertility. Among the models tested, Naïve Bayes and Logistic Regression provided the most reliable and generalizable performance. The incorporation of SHapley Additive exPlanations (SHAP) analysis enhanced the interpretability of the models, identifying polyendocrine metabolic ovarian syndrome (PMOS, previously known as polycystic ovarian syndrome—PCOS), AMH, poor ovarian reserve, menstrual cycle irregularity, systolic blood pressure, body mass index (BMI), fasting glucose, and triglycerides as the most influential predictors of female fertility. However, future studies incorporating data from multiple centres, comprising a larger, more representative population, and using more interpretable models could enhance the reliability of ML in clinical decision-making.

## 1. Introduction

Infertility is a disorder of the reproductive system characterized by the inability to conceive naturally after at least one year of regular, unprotected sex. It affects millions worldwide, with estimates suggesting that about one in six people of reproductive age will face it during their lifetime [1]. Globally, infertility affects between 12.6% and 17.5% of couples of reproductive age [2]. In India, the primary infertility rate among women of reproductive age varies from 3.9% to 16.8%, and in northeast India, it is reported at 1.44% [3,4]. Female infertility is a multifactorial disease; therefore, diagnosis is generally expensive, and accurately identifying causes is crucial for effective management of the condition. Often, diagnosing female infertility proves immensely challenging for clinicians, leading to the development of various predictive models to aid in its evaluation [5]. Female fertility is influenced by a myriad of predictors that can be categorized into demographic, anthropometric, metabolic, hormonal, and clinical parameters. Notably, age represents one of the most significant demographic factors affecting both female and male partners, with advanced maternal age associated with a decline in reproductive potential [6]. Key anthropometric measurements, including body mass index (BMI) and waist circumference, also play a crucial role in fertility outcomes [6,7]. Metabolically, fasting glucose [8], high-density lipoprotein cholesterol (HDL-C) [9] and triglyceride levels [8] have been found to significantly impact clinical pregnancy rates. Hormones, particularly anti-Müllerian hormone (AMH) [10], thyroid-stimulating hormone (TSH) [11], and serum prolactin levels [12], are also critical determinants of female fertility. Thyroid dysfunction is often associated with female infertility, as it can interfere with ovarian function, hinder implantation, cause anovulation, lead to hyperprolactinemia, and disrupt sex hormone balance [11]. Several clinical conditions may impede natural conception, and these include irregular menstrual cycles, amenorrhea, dysmenorrhea [13], the age at menarche [14], frequency of sexual intercourse [15], dyspareunia (pain in intercourse) [16], polycystic ovary [17], diminished ovarian reserve [18], uterine fibroids [19], and tubal blockage [20]. Furthermore, male semen parameters—such as semen volume, sperm concentration, total sperm count, total motile sperm, progressively motile sperm, and sperm morphology—are essential components for achieving a successful clinical pregnancy [21].

The relationship between female fertility and age is particularly pronounced, as advancing age is associated with declines in oocyte quantity and quality [22]. Research findings indicate that age-associated infertility in females is associated with increased inflammation and metabolic alterations, as evidenced by proteomic analysis of the follicular microenvironment in aged women. Additionally, a recent meta-analysis reported that women aged 35 years and older exhibit a 2.07-fold increased risk of infertility compared to younger women [23]. Another significant contributor is the BMI. Elevated BMI can disrupt the hypothalamic–pituitary–ovarian (HPO) axis by increasing adipose tissue, thereby enhancing aromatase activity and elevating estrogen levels. These hormonal alterations can lead to reduced luteinizing hormone (LH) levels, resulting in ovulatory dysfunction [24]. Moreover, high BMI is associated with menstrual irregularities, oligo-anovulation, and elevated risks of early pregnancy loss [25]. According to the National Family Health Survey-5 (NFHS-5) conducted in India from 2019 to 2021, 21.3% of women aged 15 and older were identified as having hypertension. Research indicates that elevated blood pressure—both systolic and diastolic—compromises reproductive health by impairing endothelial function, inciting chronic inflammatory responses, and disrupting hormonal balance, all of which can detrimentally affect ovulation and embryo implantation [26]. Ovarian reserve indicates the quantity and quality of a woman’s remaining oocytes. It is a key factor in evaluating fertility potential, as diminished ovarian reserve is strongly associated with compromised fertility potential [18]. Similarly, polyendocrine metabolic ovarian syndrome (PMOS), previously known as polycystic ovary syndrome (PCOS), is another common cause of infertility among women of reproductive age worldwide. It not only alters blood levels of follicle-stimulating hormone (FSH), LH, progesterone, oestradiol (E2), and sex hormone-binding globulin (SHBG), but also markedly affects the activity of the hypothalamus-pituitary-ovarian (HPO) axis and elevates gonadotropin-releasing hormone (GnRH) frequency, which ultimately impairs the fertility potential of females through disrupting follicular development and ovulation [17,27]. PMOS is often associated with elevated AMH released from primary to large antral follicles and plays a crucial role in early follicular growth. PMOS women usually have a low number of mature oocytes compared to healthy fertile females [28]. Additionally, a recent meta-analysis reported that the association of fibroids with female infertility remains unclear. However, fibroids can disrupt physiological myometrial motility in the uterus and interfere with sperm progression inside the uterus and embryo implantation, leading to impaired pregnancy [29]. On the other hand, fallopian tube blockage is a significant risk factor for female infertility because the fallopian tubes enable sperm to fertilize the ova and transfer the conceptus from the ampullary part to the uterine cavity. Blockages in one or both tubes prevent fertilization and ultimately impair implantation, resulting in infertility [30]. Furthermore, analyzing the male partner’s semen is essential for identifying the causes of infertility in the couple.

In recent years, machine learning (ML) has received extensive attention in biomedical research for its ability to develop predictive models that tailor disease management to measurable variables, thereby enabling the development of diagnostic models [31]. The reproducibility of ML approaches involves standardized feature extraction and transparent model training. For example, deep learning models are gaining attention for their ability to analyze complex nonlinear patterns in multidimensional datasets [32,33]. ML provides a robust analytical framework for identifying complex associations among biological, clinical, metabolic, and lifestyle factors. Unlike conventional statistical analysis, the ML approaches capture nonlinear interactions and latent patterns among reproductive variables. ML thus represents a promising tool for precision in reproductive medicine, offering improved predictive performance, enhanced decision-making, and the potential to optimise fertility management and reproductive outcomes [34,35].

Therefore, in the present study, we included all 28 aforementioned predictors and used ML approaches to validate fertility diagnostic models, thereby more accurately evaluating female infertility and improving the effectiveness, cost-efficiency, and precision of infertility management.

## 2. Materials and Methods

### 2.1. Study Design and Data Collection

This retrospective analytical study aimed to categorize female fertility status based on various clinical, hormonal, metabolic, and reproductive health parameters using ML methods. It analyzed 101 samples, including both fertile and infertile cases. The data, obtained from patient records at a tertiary fertility healthcare centre, encompassed female-specific parameters as well as relevant semen characteristics of male partners to capture the multifactorial aspects of infertility. Each sample was labelled as either fertile (coded as 1) or infertile (coded as 0) based on clinical diagnoses and treatment outcomes recorded for each participant. Sample size was calculated using the following formula described by Liang and Colleagues [36].

Z_α/2_^2^P(1 − P)/E^2^,

where (Z_α/2_ = 1.64, P = 1.44%, E = 2%), with an allowable error of 2% and a 90% confidence level, the calculated sample size was 93.

### 2.2. Study Variables

A total of 28 predictor variables were selected, grouped into categories: demographic (patient age, male partner age), anthropometric (BMI, waist circumference), and blood pressure (systolic and diastolic blood pressure). Menstrual and gynaecological history included menstrual cycle irregularity, amenorrhea, period cramps, age at first period, and pain during intercourse. Clinical conditions encompassed polycystic ovaries, poor ovarian reserve, tubal blockage, and fibroids. Hormonal profile included AMH, TSH, and prolactin. The metabolic profile included fasting glucose, HDL-C, and triglycerides. Semen parameters included semen volume, sperm concentration, total sperm count, total motile sperm, progressive motile sperm, and sperm morphology. Sexual behaviour was evaluated by the frequency of intercourse per week. All features were either continuous or binary-coded as required for analysis.

### 2.3. Data Preprocessing

Prior to analysis, all continuous variables were checked for missing values, outliers, and distribution characteristics. Since no missing values were detected, complete case analysis was employed. Continuous variables were standardized using z-score normalization to ensure a uniform scale across features for the ML algorithms. Z = (x − μ)/σ, where Z is the standardized value, x is the original value, *μ*, is the mean of the variable, and σ is the standard deviation, was used. Categorical variables were binary encoded (0 = absent, 1 = present). The normality of each predictor was assessed using the Shapiro–Wilk test, which guided the selection of appropriate statistical tests. The study dataset comprised 101 observations, including 75 infertile and 26 fertile cases. A total of 28 predictor variables were initially considered, resulting in a feature-to-sample ratio of 3.61.

### 2.4. Exploratory Data Analysis and Statistical Testing

Descriptive statistics were calculated as mean ± standard deviation (SD) for variables. To compare characteristics between fertile and infertile groups, independent *t*-tests were used for normally distributed continuous variables. Mann–Whitney U tests were applied to non-normally distributed variables. Pearson correlation analysis was performed to evaluate relationships among variables, including correlations with fertility status. Visual exploration analysis included Kernel Density Estimation (KDE) plots for each feature, stratified by fertility group, and heatmaps of the correlation matrix to illustrate interdependencies.

### 2.5. Multicollinearity Assessment and Sensitivity Analysis

Given the relatively large number of predictors relative to the sample size, multicollinearity among variables was assessed using Variance Inflation Factors (VIFs). Variables exhibiting severe multicollinearity and variables with extremely low prevalence were evaluated through sensitivity analysis. A reduced predictor set was subsequently developed by removing highly correlated variables and predictors that occurred very infrequently in the dataset. This process reduced the number of predictors from 28 to 20. The predictive performance of ML models was subsequently compared between the full and reduced datasets to assess model robustness and the influence of redundant predictors.

### 2.6. Machine Learning Model Development and Validation

The fertility classification was formulated as a supervised binary classification problem. Seven ML algorithms were evaluated, including Logistic Regression, Random Forest, Decision Tree, Support Vector Machine, Naïve Bayes, K-Nearest Neighbour and Extreme Gradient Boosting (XGBoost). To ensure reliable estimation of model performance and minimize overfitting, stratified 10-fold cross-validation was employed. Stratification preserved the class distribution of fertile and infertile cases within each fold. For each iteration, nine folds were used for training and one for validation. Data standardization was performed independently within each training fold using an ML pipeline. This procedure prevented information leakage from the validation data into the training process and ensured an unbiased assessment of model performance. Hyperparameter optimization was performed using Grid Search combined with stratified 10-fold cross-validation. Separate parameter grids were defined for each ML algorithm, and the optimal hyperparameter combination was selected based on the highest cross-validated area under the receiver operating characteristic curve (AUROC) (Supplementary Table S1). In addition to the full predictor set, three separate analyses were conducted using: (i) female-related variables only, (ii) male-related variables only, and (iii) the combined predictor set. This comparison was performed to evaluate the relative contribution of female and male fertility factors to classification performance.

### 2.7. Evaluation Metrics

The models were evaluated using multiple performance metrics, such as: (i) accuracy (proportion of correctly predicted samples), (ii) precision (ability of the model to identify only relevant cases—true positives/predicted positives), (iii) recall (sensitivity—ability to find all relevant cases as true positives/actual positives), (iv) F1-Score (harmonic mean of precision and recall), and (v) area under the curve (AUC) to reflect the model’s discriminative ability across thresholds (vide formula given below).

$$AUC=\int_{0}^{1}TPR(FPR)dFPR$$

To evaluate model stability and potential overfitting, both training AUROC and cross-validated AUROC were recorded. An overfitting gap was calculated as the difference between the training AUROC and the validation AUROC. Smaller gap values indicated stronger generalization performance and greater model robustness. ROC curves were generated using the mean performance obtained across the 10 cross-validation folds.

### 2.8. Feature Importance and Model Interpretability

To identify the variables contributing most strongly to fertility classification, feature importance analysis was performed using the Random Forest model. Feature importance scores provide a global assessment of each predictor’s relative contribution to the classification process and facilitate the identification of clinically relevant variables. To further enhance model interpretability, SHapley Additive exPlanations (SHAP) analysis was applied to the Random Forest model. SHAP values quantify the contribution of individual predictors to model predictions based on principles derived from cooperative game theory. Unlike conventional feature importance measures, SHAP provides both global and local interpretations by explaining how individual variables influence predictions for each observation. By linking model outputs to clinically meaningful reproductive, hormonal, metabolic, and semen-related variables, SHAP analysis improves transparency and supports the interpretation of ML predictions in a clinical context. This approach enhances the potential utility of ML models as interpretable decision-support tools in reproductive health research [37].

## 3. Results

### 3.1. Exploratory Data Analysis

**Figure 1:**
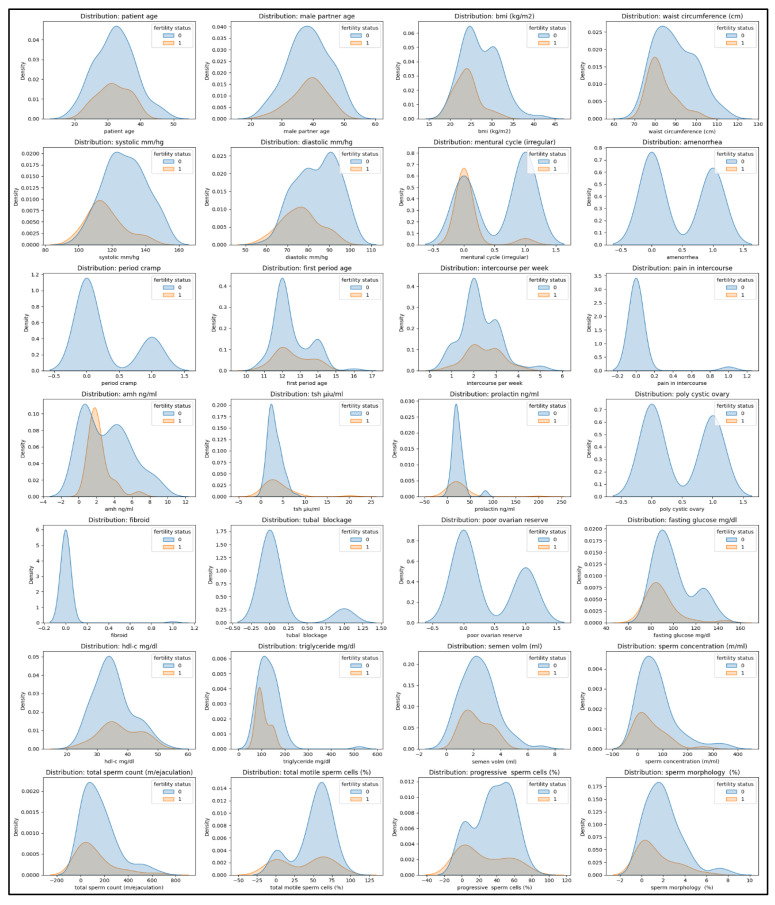
Kernel Density Estimation (KDE) plots for 28 clinical, hormonal, and metabolic predictors, stratified by fertility status (0 = infertile, 1 = fertile). The plots visually compare the distributions of each variable between fertile and infertile individuals, revealing key differences in body mass index (BMI), blood pressure, reproductive disorders, metabolic markers, and semen parameters.

The descriptive analysis of all 28 predictors revealed considerable variation across demographic, clinical, hormonal, and semen parameters in the study population. The mean age of female participants was 32.08 ± 5.64 years, and that of their male partners was 38.59 ± 6.24 years. The average BMI was 26.44 ± 4.33 kg/m^2^, and the mean waist circumference measured 88.03 ± 9.55 cm. Systolic and diastolic blood pressures were 124.75 ± 13.05 mmHg and 82.18 ± 10.15 mmHg, respectively. Regarding menstrual characteristics, 45% of participants had irregular cycles (0.45 ± 0.50), while 34% reported amenorrhea (0.34 ± 0.47) and 20% experienced period cramps (0.20 ± 0.40). The average age at menarche was 12.52 ± 0.97 years, and the frequency of sexual activity was 2.32 ± 0.82 intercourses/week. Pain during intercourse was relatively rare (0.03 ± 0.17). Hormonal profiles showed mean AMH levels of 2.93 ± 2.39 ng/mL, TSH of 3.31 ± 2.34 µIU/mL, and prolactin of 24.54 ± 22.06 ng/mL. Clinical diagnoses indicated that 35% had polycystic ovaries (0.35 ± 0.48), 28% had poor ovarian reserve (0.28 ± 0.45), 10% had tubal blockage (0.10 ± 0.30), and fibroids were present in 1% of cases (0.01 ± 0.10). Biochemical indices showed an average fasting glucose level of 97.56 ± 17.48 mg/dL, HDL-C of 35.73 ± 6.32 mg/dL, and triglycerides of 125.52 ± 53.12 mg/dL. Semen analysis parameters indicated a mean semen volume of 2.34 ± 1.23 mL, sperm concentration of 69.87 ± 73.66 million/mL, and total sperm count per ejaculation of 148.86 ± 153.95 million. Total motile sperm cells made up 47.75 ± 27.22%, with 33.00 ± 23.56% showing progressive motility. The average sperm morphology score was 1.83 ± 1.72%, suggesting a dominance of sperm morphological defects. Figure 1 provides a visual comparison of the distributions of the variables between the two groups (i.e., fertile and infertile), highlighting potentially discriminative features. Moreover, to identify differences in clinical, hormonal, and metabolic characteristics between fertile and infertile individuals, the normality of each of the 28 predictor variables was evaluated first using the Shapiro–Wilk test. Variables that met the normality assumption were analyzed with independent samples *t*-tests, while those violating normality were assessed with the non-parametric Mann–Whitney U test. A significance level of *p*  <  0.05 was used to determine statistical significance.

Among the variables analyzed, patient age (*p* = 0.7164) and male partner Age (*p* = 0.9583) displayed a normal distribution and were evaluated with *t*-tests. Neither variable showed significant differences between fertile and infertile groups, suggesting that age alone did not differentiate the groups in this population. In contrast, several other variables showed significant group differences when assessed with the Mann–Whitney U test. Anthropometric and cardiovascular parameters including BMI (*p* = 0.0004), waist circumference (*p* = 0.0021), systolic blood pressure (*p* = 0.0001), and diastolic blood pressure (*p* = 0.0006) were significantly higher in infertile individuals. These findings suggest that increased adiposity and elevated blood pressure may be associated with reproductive dysfunction. Reproductive health indicators showed robust associations with fertility status. Variables including menstrual cycle irregularity (*p* < 0.0001), amenorrhea (*p* < 0.0001), and period cramps (*p* = 0.0035) were significantly more prevalent in the infertile group. In addition, structural or functional reproductive abnormalities such as polycystic ovaries (*p* < 0.0001) and poor ovarian reserve (*p* = 0.0003) also showed significant differences, aligning with established clinical evidence linking these conditions to female infertility. For biochemical and metabolic parameters, fasting glucose (*p* = 0.0006) and triglyceride levels (*p* = 0.0034) were significantly higher in infertile participants. These metabolic markers may reflect underlying insulin resistance or dyslipidemia, both of which are known to negatively affect fertility. Among male counterpart-related variables, sperm concentration (*p* = 0.0494) and sperm morphology (*p* = 0.0384) were significantly different between groups, with infertile individuals showing poorer semen quality. These results underscore the importance of considering both male and female contributions in fertility diagnostics.

**Figure 2:**
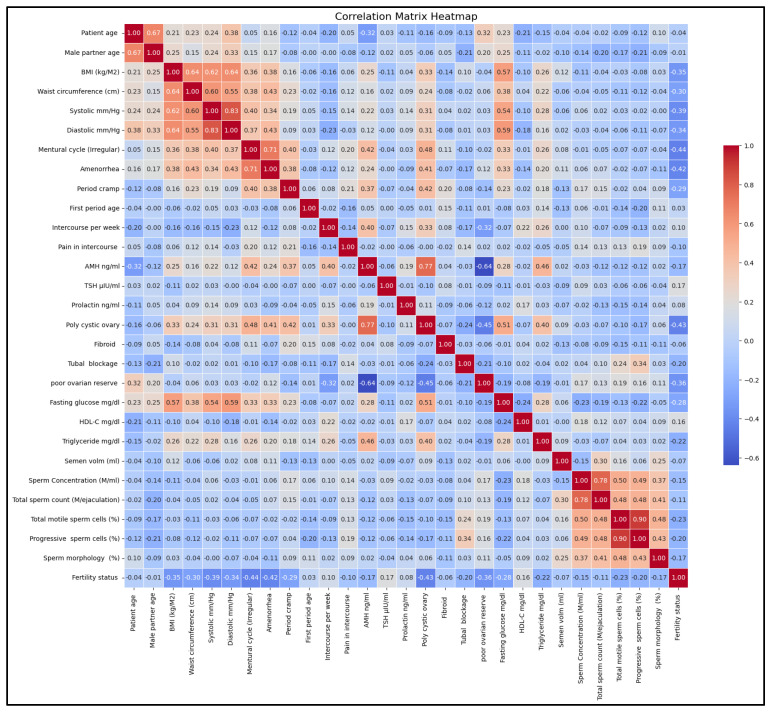
Correlation matrix heatmap showing Pearson correlation coefficients among all 28 predictor variables and fertility status (coded as 0 = infertile, 1 = fertile). Strong positive correlations are indicated in red, negative correlations in blue, with colour intensity reflecting the strength of the correlation. Notable relationships were observed among metabolic parameters, hormonal markers, and semen quality indicators.

Conversely, several features did not demonstrate statistically significant differences between the groups. These included factors such as age at the first period, frequency of intercourse, pain experienced during intercourse, serum levels of AMH, TSH, and prolactin, as well as HDL-C, semen volume, total sperm count, total motile sperm cells, and progressively motile sperm cells. A comprehensive Pearson correlation analysis was performed on all clinical, hormonal, metabolic, and reproductive variables, including the binary outcome of fertility status. The correlation matrix presented in Figure 2 summarizes these pairwise correlations, highlighting several important trends and associations.

Firstly, notable clustering was observed among metabolic indicators. Specifically, BMI showed a positive correlation with waist circumference (r = 0.57), systolic blood pressure (r = 0.41), and diastolic blood pressure (r = 0.38). This suggests a shared underlying cardiometabolic profile. These variables are clinically known to be associated with obesity-related reproductive disorders, and their interrelatedness may indicate broader systemic dysregulation in patients experiencing infertility. In addition, hormonal and reproductive health markers exhibited significant relationships. PMOS was strongly correlated with menstrual irregularity (r = 0.71) and amenorrhea (r = 0.77), consistent with the clinical features of PMOS. Moreover, AMH showed a positive correlation with the presence of polycystic ovaries (r = 0.37), reinforcing AMH’s relevance as a biomarker for diagnosing PMOS and assessing ovarian reserve. On the other hand, strong internal correlations were found among male fertility parameters, including sperm concentration, total sperm count, total motile sperm cells, and progressively motile sperm cells, with correlation coefficients frequently exceeding 0.6. These findings reflect the expected coherence among semen quality indicators and support their combined utility in evaluating male fertility potential. Lastly, correlations related to fertility status indicated that certain factors may be more discriminative. Negative associations were identified with waist circumference (r = −0.35), systolic blood pressure (r = −0.30), poor ovarian reserve (r = −0.28), and triglycerides (r = −0.33), suggesting a connection between these metabolic factors and female infertility. In contrast, weak positive correlations were observed between fertility and sperm morphology (r = 0.17) and AMH (r = 0.14), although they had limited discriminative power when considered individually.

### 3.2. Multicollinearity Assessment

Multicollinearity among predictor variables was assessed using VIFs. The analysis revealed substantial multicollinearity among several demographic, anthropometric, cardiovascular, metabolic, and semen-related variables (Supplementary Table S2). The highest VIF values were observed for systolic blood pressure (VIF = 513.33), diastolic blood pressure (VIF = 390.98), waist circumference (VIF = 201.99), age at first menstruation (VIF = 116.40), BMI (VIF = 113.12), patient age (VIF = 102.28), and male partner age (VIF = 97.99). Elevated VIF values were also detected for fasting glucose, HDL-C, total motile sperm cells, and progressively motile sperm cells, indicating considerable redundancy among several predictors. The observed multicollinearity is biologically plausible because many of these variables represent related physiological processes. For example, BMI, waist circumference, blood pressure, fasting glucose, and lipid parameters collectively reflect metabolic health and are frequently associated with obesity-related reproductive disorders. Similarly, sperm concentration, total sperm count, total sperm motility cells, and progressively motile sperm cells represent interconnected measures of semen quality and therefore exhibit strong interdependence. To evaluate the effect of multicollinearity on model stability, a sensitivity analysis was performed. Variables exhibiting severe multicollinearity and predictors with extremely low prevalence were removed from the original predictor set. Specifically, waist circumference, diastolic blood pressure, male partner age, amenorrhea, progressively motile sperm cells, total sperm count, pain during intercourse, and fibroid status were excluded, reducing the number of predictors from 28 to 20 (Supplementary Table S3). Subsequent VIF analysis demonstrated a substantial reduction in multicollinearity while preserving clinically relevant reproductive, hormonal, metabolic, and semen-related variables. The reduced predictor set was subsequently used for model development and validation. Comparative analyses between the full and reduced datasets revealed only minor changes in predictive performance, indicating that the proposed ML framework was robust to predictor redundancy and multicollinearity.

### 3.3. Sensitivity Analysis

To evaluate the robustness of the proposed ML framework and assess the effects of multicollinearity and predictor redundancy, a sensitivity analysis was conducted with a reduced set of predictors. The original dataset contained 28 predictors, several of which showed substantial multicollinearity as indicated by VIF analysis. In addition, some variables showed extremely low prevalence within the study population. Consequently, waist circumference, diastolic blood pressure, male partner age, amenorrhea, progressively motile sperm cells, total sperm count, pain during intercourse, and fibroid status were removed, resulting in a reduced dataset comprising 20 predictors. The predictive performance of all ML models was subsequently re-evaluated using stratified 10-fold cross-validation (Table 1). The results demonstrate that model performance remained largely stable following variable reduction. Naïve Bayes achieved the highest classification performance, with an accuracy of 98.0%, precision of 97.5%, recall of 95.0%, F1-score of 95.2%, and AUROC of 0.993. Logistic Regression and Support Vector Machine also maintained strong discriminative performance, achieving AUROC values of 0.976 and 0.965, respectively. To further investigate model stability, overfitting was assessed by comparing the training AUROC with the cross-validated AUROC values. Naïve Bayes exhibited perfect agreement between training and validation performance (Gap = 0.000), indicating excellent generalization ability. Logistic Regression (Gap = 0.023), Support Vector Machine (Gap = 0.033), Random Forest (Gap = 0.036), and K-Nearest Neighbour (Gap = 0.034) also demonstrated low overfitting risk and stable predictive performance. In contrast, the Decision Tree exhibited substantial overfitting (Gap = 0.219), while XGBoost showed moderate overfitting (Gap = 0.065) despite maintaining strong classification performance.

### 3.4. Comparative Analysis of Female, Male, and Combined Predictors

To evaluate the relative contributions of female and male fertility factors, separate ML analyses were performed using female-only, male-only, and combined predictor sets. The performance of the best-performing model from each dataset is presented in Table 2. The female-only dataset demonstrated excellent predictive performance, with Naïve Bayes achieving an AUROC of 0.993 and an accuracy of 98.0%. Logistic Regression also exhibited outstanding discrimination (AUROC = 0.995), indicating that female clinical, hormonal, metabolic, and reproductive health variables contain substantial predictive information for fertility classification. In contrast, the male-only dataset produced considerably lower predictive performance. The best-performing model, Naïve Bayes, achieved an AUROC of only 0.614 and an accuracy of 72.1%. Similar reductions in performance were observed across all evaluated classifiers, suggesting that semen characteristics alone were insufficient for reliable fertility classification within the current dataset. The combined dataset yielded the best overall performance, with Naïve Bayes achieving 98.0% accuracy and an AUROC of 0.993. However, the improvement over the female-only dataset was modest. This finding indicates that the majority of predictive information originated from female-related clinical and hormonal characteristics, while semen parameters provided comparatively limited additional discriminatory value.

### 3.5. Receiver Operating Characteristic (ROC) Curve Analysis

**Figure 3:**
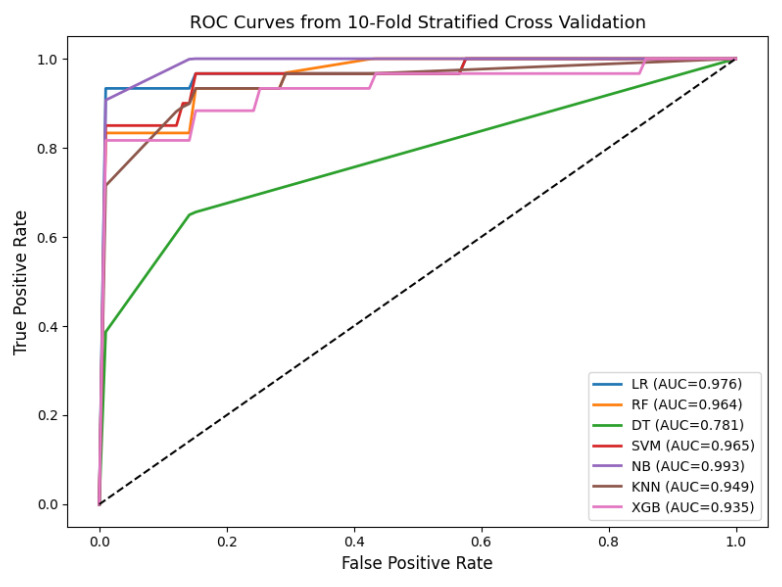
Receiver operating characteristic (ROC) curves obtained from stratified 10-fold cross-validation for the evaluated machine learning models using the reduced predictor dataset. Naïve Bayes achieved the highest discriminative performance (area under the ROC, i.e., AUROC = 0.993), followed by Logistic Regression (AUROC = 0.976), Support Vector Machine (AUROC = 0.965), and Random Forest (AUROC = 0.964).

The discriminative performance of the evaluated ML models was further assessed using ROC curves generated from stratified 10-fold cross-validation (Figure 3). The ROC curves illustrate the trade-off between sensitivity and specificity across different classification thresholds and provide a comprehensive measure of model performance via the AUROC. Among the evaluated models, Naïve Bayes achieved the highest discriminative performance with an AUROC of 0.993, followed by Logistic Regression (AUROC = 0.976), Support Vector Machine (AUROC = 0.965), and Random Forest (AUROC = 0.964). These models exhibited ROC curves that remained near the upper-left corner of the plot, indicating excellent classification performance and strong discrimination between fertile and infertile individuals. K-Nearest Neighbour also demonstrated satisfactory performance with an AUROC of 0.949, while XGBoost achieved an AUROC of 0.935. Although XGBoost maintained strong predictive performance, its ROC curve was slightly inferior to those of Naïve Bayes, Logistic Regression, Support Vector Machine, and Random Forest. The Decision Tree exhibited the lowest discriminative performance (AUROC = 0.781), consistent with the overfitting behaviour observed during the sensitivity analysis.

### 3.6. Feature Importance Analysis

**Figure 4:**
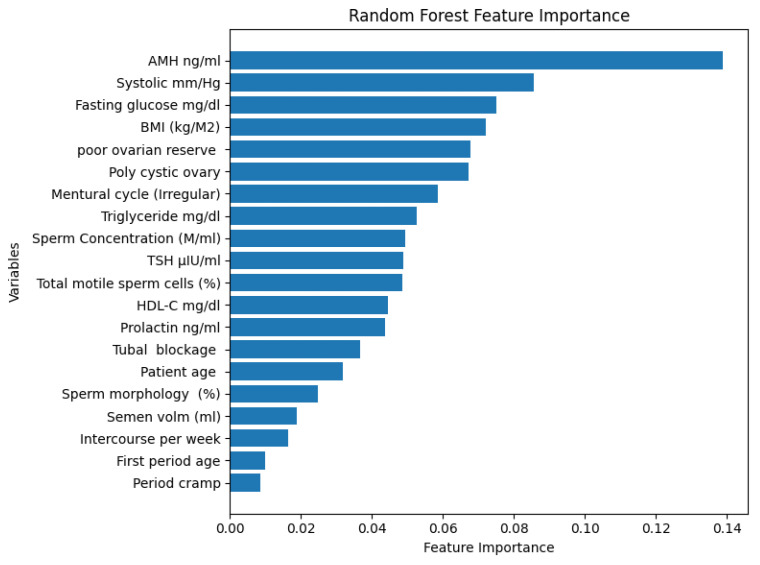
Random Forest feature importance ranking of predictors used for fertility classification. Anti-Müllerian hormone (AMH), systolic blood pressure, fasting glucose, body mass index (BMI), poor ovarian reserve, and polyendocrine metabolic ovarian syndrome (PMOS)—previously called polycystic ovarian syndrome (PCOS) were the most influential variables.

To identify the predictors most strongly associated with fertility classification, a feature importance analysis was performed using the Random Forest model (Figure 4). The results indicated that AMH was the most influential predictor, followed by systolic blood pressure, fasting glucose, BMI, poor ovarian reserve, and PMOS. The menstrual cycle strongly contributes to systolic blood pressure, fasting glucose, BMI, triglycerides, and HDL-C, suggesting that metabolic health plays a significant role in fertility outcomes. Among the male-related variables, irregularity was observed in sperm concentration, total motile sperm cells, triglyceride levels, TSH, and total motile sperm cells.

### 3.7. Model Explainability Using SHapley Additive exPlanations (SHAP) Analysis

**Figure 5:**
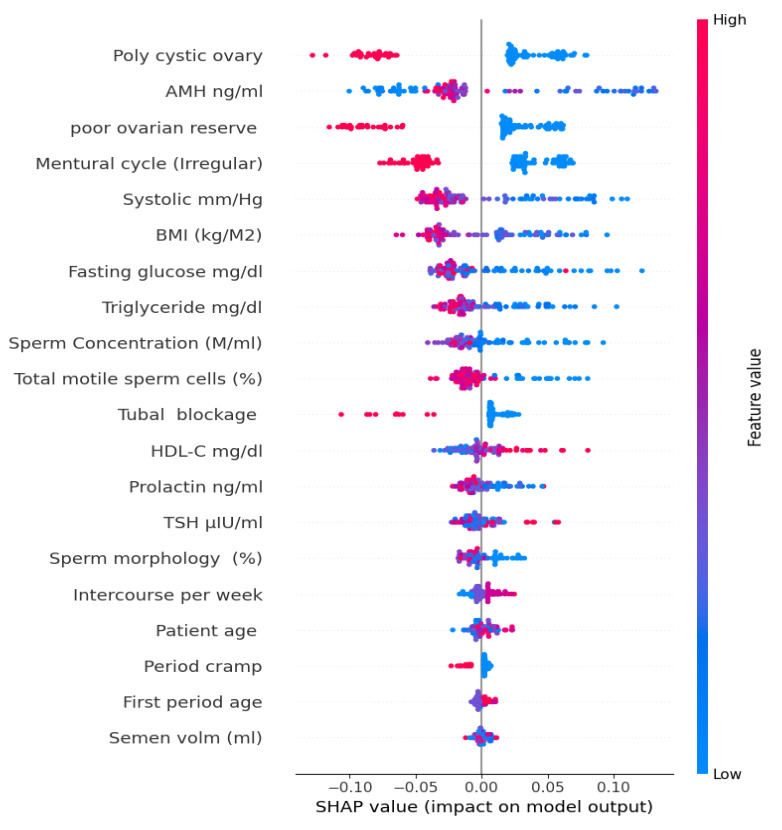
SHapley Additive exPlanations (SHAP) summary (beeswarm) plot illustrating the contribution of individual predictors to fertility classification. Features are ordered by their average impact on model output, with colour representing feature values and SHAP values indicating the direction and magnitude of each predictor’s influence.

To further improve model interpretability, SHAP values were calculated using a Random Forest. The SHAP summary plot (Figure 5) provides a global overview of feature contributions and illustrates the direction and magnitude of each feature’s effect on model predictions. The SHAP analysis identified PMOS, AMH, poor ovarian reserve, menstrual cycle irregularity, systolic blood pressure, BMI, fasting glucose, and triglycerides as the most influential predictors of fertility status. These findings were largely consistent with the Random Forest feature importance rankings, thereby supporting the robustness of the identified predictors.

Higher values of PMOS, poor ovarian reserve, menstrual cycle irregularity, fasting glucose, BMI, triglycerides, and systolic blood pressure generally contributed to increased probability of infertility. In contrast, higher AMH levels were associated with reduced infertility risk, reflecting the established role of ovarian reserve in female reproductive health. Similarly, favourable semen parameters, including higher sperm concentration and total motile sperm cells, contributed positively to fertility classification, although their overall influence was less pronounced than that of female reproductive and metabolic variables. The SHAP beeswarm plot further demonstrated considerable heterogeneity in the influence of individual predictors across participants, indicating that fertility status is determined by complex interactions among reproductive, hormonal, metabolic, and semen-related characteristics. The predominance of female reproductive variables within the SHAP rankings supports the findings of the comparative predictor analysis, which showed that female-only models achieved substantially higher predictive performance than male-only models. Collectively, the SHAP analysis provides clinically interpretable evidence that ovarian reserve, endocrine function, metabolic health, and menstrual characteristics are the principal determinants of fertility classification within the present study population.

## 4. Discussion

The present study undertook a comprehensive examination of the application of ML techniques to identify predictors of female fertility. By leveraging a diverse array of clinical, hormonal, and biochemical characteristics, along with semen analysis of male counterparts, the study aimed to enhance our understanding of multifactorial female infertility. The approach incorporated exploratory statistical methods, a detailed evaluation of model performance, an analysis of feature importance, and the application of model explainability techniques. This combination provided a robust framework not only for assessing the efficacy of ML models but also for elucidating the complex interactions among the variables influencing female fertility outcomes.

The results of the exploratory analysis highlight important clinical patterns among infertile individuals. Notably, infertile females exhibited significantly higher BMI, waist circumference, systolic and diastolic blood pressures, fasting glucose, and triglyceride levels. These findings are consistent with a previous study from the United States, where BMI was identified as a significant predictor of female infertility, which impairs pregnancy development [38]. Similarly, higher systolic and diastolic pressures are related to compromised egg quality, and hypertensive females often suffer from metabolic diseases resulting in excessive estrogen production, leading to infertility [39]. Similar to the present study, Zhurang and his team also reported higher levels of triglyceride and fasting glucose in infertile females compared to healthy fertile females [8]. The metabolic factors impair female fertility through multiple pathways. High insulin levels act on the ovaries, increasing androgen production by acting upon theca cells. Additionally, it reduces SHBG production, thus increasing the amount of free androgen circulating in the bloodstream [40]. Moreover, strong correlations between BMI, blood pressure, and waist circumference confirm the clustering of cardiometabolic risk factors in the infertile group. These risk factors are often associated with inflammation in the reproductive organs of females, via elevating pro-inflammatory cytokines such as tumour necrosis factor-alpha (TNF-α), interleukin-6 (IL-6), interleukin- 1 beta (IL-1β), compromising oocyte quality, impairing folliculogenesis, disrupting hormone production, and ultimately contributing to female infertility [41]. Hormonal factors also played a significant role in distinguishing fertility status. The prevalence of polycystic ovaries and poor ovarian reserve was substantially higher among infertile women, consistent with known etiologies of anovulation and subfertility. Menstrual cycle irregularity and amenorrhea were also prominent features, particularly in women with PMOS, as supported by strong inter-variable correlations (e.g., PMOS with menstrual irregularity, *r* = 0.71; and amenorrhea, *r* = 0.77). Semen parameters, particularly sperm concentration and morphology, also emerged as significant contributors to fertility classification. Although semen volume and total sperm count did not differ significantly, the qualitative aspects of semen (e.g., morphology) were more indicative of male subfertility in this sample.

The predictive performance of the seven utilized ML models was assessed by using stratified 10-fold cross-validation on the reduced predictor dataset (Table 1). Naïve Bayes achieved the highest overall performance among the classifiers evaluated, with an accuracy of 98.0%, precision of 97.5%, recall of 95.0%, F1-score of 95.2%, and AUROC of 0.993. In contrast, the Logistic Regression also showed outstanding discriminative competence (AUROC = 0.976) and strong classification performance, followed by Support Vector Machine (AUROC = 0.965) and Random Forest (AUROC = 0.964). K-Nearest Neighbour achieved satisfactory predictive performance (AUROC = 0.949), whereas Decision Tree exhibited the lowest discriminative ability (AUROC = 0.781). Model stability was additionally assessed by comparing the training and cross-validated AUROC values. The Naïve Bayes showed impeccable consensus between training and validation performance (Gap = 0.000), which showed excellent generalization. Logistic Regression (Gap = 0.023), Support Vector Machine (Gap = 0.033), Random Forest (Gap = 0.036), and K-Nearest Neighbour (Gap = 0.034) also showed reduced overfitting risk and have stable predictive performance. On the other hand, Decision Tree exhibited substantial overfitting (Gap = 0.219), while XGBoost showed moderate overfitting (Gap = 0.065) despite maintaining strong predictive performance. These findings showed that simple ML algorithms such as Naïve Bayes and Logistic Regression perform better than complex algorithms. Simpler models effectively capture latent patterns in the data and minimize the risk of overfitting, resulting in more reliable performance. Similar to the findings of the present study, Huang and colleagues reported that ML-based models are more effective than conventional statistical approaches at capturing non-linear relationships and complex interactions in biological datasets, thereby achieving better performance [34]. These findings are further supported by the ROC curve produced from the stratified 10-fold cross-validation (Figure 3). The highest AUROC values were achieved by Naïve Bayes and Logistic Regression, and their ROC curves lay closer to the upper-left corner of the plot, indicating strong discrimination between fertile and infertile individuals. This ROC analysis validates the superior generalization performance of these models and confirms their suitability for fertility classification. The Random Forest model-based feature importance analysis (Figure 4) revealed that AMH, systolic blood pressure, fasting glucose, BMI, poor ovarian reserve, and PCOS were the most influential predictors of fertility status. Moreover, significant differences were observed for menstrual cycle irregularity, triglycerides, sperm concentration, TSH, and total sperm motility. The multifactorial nature of infertility was observed from predominance of hormonal, metabolic and reproductive variables and underscores the significance of integration of an array of clinical indicators in predictive models. Model interpretability was further improved by SHAP analysis of the Random Forest model (Figure 5). The SHAP summary (beeswarm) highlighted that AMH, PMOS, poor ovarian reserve, menstrual cycle irregularity, fasting glucose, BMI, systolic blood pressure, sperm concentration and total sperm motility exerted a strong influence on model predictions. This study recommended that semen analysis of the male partner should be the initial step in evaluating an infertile couple. Evaluation of the female partner should begin if the male partner’s semen analysis is within the normal range.

However, the present study is not free of limitations. The small sample size posed challenges to model development and evaluation, thereby increasing the risk of overfitting. Another limitation was the lack of external validation using independent datasets from different clinical settings. However, internal validation provided an initial assessment of model robustness. Therefore, future studies should aim to validate the present study’s findings in independent external cohorts using larger, more diverse datasets, explore longitudinal data to understand causal relationships, and investigate ensemble approaches that combine the strengths of multiple models to achieve more accurate fertility diagnostics. Despite the limitations, the present study provides significant contributions to the growing field of artificial intelligence applications in reproductive medicine. The study demonstrated the feasibility of applying ML methods to integrate metabolic factors, reproductive hormones, and seminal characteristics to predict female fertility. Although limited in size, the present study provides valuable insights into the complex interactions among the variables examined. Moreover, the use of multiple ML algorithms and comparative performance assessment strengthens the findings and can serve as a benchmark for further studies in reproductive medicine.

## 5. Conclusions

The present study demonstrates that properly evaluated and interpreted ML models can effectively classify female fertility using multidimensional clinical, hormonal, metabolic, and reproductive health indicators. Additional validation analyses, including multicollinearity assessment, sensitivity analysis, stratified 10-fold cross-validation, and overfitting evaluation, confirmed the robustness and stability of the proposed classification framework. Among the evaluated models, Naïve Bayes and Logistic Regression exhibited the strongest generalization performance, achieving high predictive accuracy while maintaining minimal train–validation performance gaps. Comparative analyses using female-only, male-only, and combined predictor sets revealed that female reproductive, hormonal, and metabolic characteristics contributed substantially more predictive information than male semen parameters. Although the combined dataset achieved the highest overall classification performance, the improvement over female-only models was modest, indicating that the primary predictive signal originated from female-related factors. Feature importance and SHAP analyses consistently identified AMH, PMOS, poor ovarian reserve, menstrual cycle irregularity, fasting glucose, body mass index, and blood pressure measures as the most influential predictors of fertility status. Although limited by a modest sample size and the absence of external validation, the study demonstrated the potential of ML algorithms in reproductive medicine by integrating metabolic, hormonal, and seminal biomarkers to predict fertility. However, future studies incorporating data from multiple centres, comprising a larger, more representative population, and using more interpretable models could enhance the reliability of ML in clinical decision-making.

## Figures and Tables

**Table 1:** Performance and overfitting assessment of machine learning (ML) models using the reduced predictor set. AUROC: area under the receiver operating characteristic curve.

Model	Accuracy	Precision	Recall	F1	Calculated Value of AUROC	Training Value of AUROC	Gap
Linear Regression	0.940	0.900	0.883	0.878	0.976	0.999	0.023
Random Forest	0.900	0.800	0.650	0.697	0.964	1.000	0.036
Decision Tree	0.851	0.758	0.633	0.672	0.781	1.000	0.219
Support Vector Machine	0.921	0.942	0.767	0.816	0.965	0.998	0.033
Naïve Bayes	0.980	0.975	0.950	0.952	0.993	0.993	0.000
K-Nearest Neighbour	0.920	0.883	0.833	0.838	0.949	0.983	0.034
Extreme Gradient Boosting (XGB)	0.910	0.767	0.717	0.733	0.935	1.000	0.065

**Table 2:** Performance comparison of the best-performing models across female-only, male-only, and combined predictor sets.

Dataset	Best Model	Accuracy	Precision	Recall	F1	Area Under the Receiver Operating Characteristic Curve (AUROC)
Female-only	Naïve Bayes	0.980	0.975	0.952	0.952	0.993
Male-only	Naïve Bayes	0.721	0.417	0.400	0.400	0.614
Combined	Naïve Bayes	0.980	0.975	0.952	0.952	0.993

## Data Availability

All the data relevant to the present study are incorporated in the manuscript.

## Supplementary Materials

The following supporting information can be downloaded at: https://www.mdpi.com/article/10.3390/bioengineering13070782/s1, Table S1: Optimal Hyperparameter Settings and Cross-Validated Area Under the Receiver Operating Characteristic Curve (AUROC) Values Obtained Through Grid Search; Table S2: Multicollinearity Assessment Using Variance Inflation Factors (VIFs); Table S3: Variables Removed During Sensitivity Analysis.

## Abbreviations

The following abbreviations are used in this manuscript:

AMH	Anti-Müllerian Hormone
AUROC	Area Under the Receiver Operating Characteristic Curve
BMI	Body Mass Index
E2	Estradiol
FSH	Follicle-Stimulating Hormone
HDL	High-Density Lipoprotein
HPO	Hypothalamic–Pituitary–Ovarian
HDL-C	High-Density Lipoprotein Cholesterol
IL-1β	Interleukin-1 beta
IL-6	Interleukin-6
KDE	Kernel Density Estimation
KNN	K-Nearest Neighbors
LR	Logistic Regression
LH	Luteinizing Hormone
ML	Machine Learning
NFHS-5	National Family Health Survey-5
PCOS	Polycystic Ovary Syndrome
RF	Random Forest
SHBG	Sex Hormone-Binding Globulin
SHAP	SHapley Additive exPlanations
SD	Standard Deviation
SVM	Support Vector Machine
TSH	Thyroid-Stimulating Hormone
TNF-α	Tumour Necrosis Factor-alpha
