# Supplementary material for: Application of Machine Learning Algorithms for Evaluating Predictors and Developing Diagnostic Models for Female Infertility Classification
Source: Bioengineering (Basel). 2026 Jul 7;13(7):782. doi: 10.3390/bioengineering13070782 (PMC13403689; doi:10.3390/bioengineering13070782)
Supplement: Supplementary file 1 [file bioengineering-13-00782-s001.zip › bioengineering-4366916-supplementary.pdf]

Supplementary Table S1. Optimal Hyperparameter Settings and Cross-Validated Area Under the Receiver Operating Characteristic Curve (AUROC) Values Obtained Through Grid Search.

Model	Optimal Hyperparameter Settings	Best AUROC
Logistic Regression	C = 1, penalty = L2, max_iter = 1000	0.976
Random Forest	n_estimators = 100, max_depth = 10, min_samples_split = 2	0.964
Decision Tree	criterion = entropy, max_depth = 5, min_samples_split = 2	0.944
Support Vector Machine	C = 1, gamma = scale, kernel = RBF	0.965
Naïve Bayes	var_smoothing = 1.05×10^{-7}	0.998
K-Nearest Neighbour	n_neighbors = 11, weights = distance	0.967
XGBoost	n_estimators = 50, max_depth = 3, learning_rate = 0.10, subsample = 1.00	0.970

Hyperparameter tuning using Grid Search demonstrated that relatively simple models achieved performance comparable to or better than more complex algorithms presented in the table. Among the evaluated classifiers, Naïve Bayes achieved the highest cross-validated AUROC (0.998), followed by Logistic Regression (0.976) and XGBoost (0.970). The optimized XGBoost model required only 50 trees with a maximum depth of 3, indicating that a relatively simple boosting structure was sufficient for the present dataset. Similarly, Logistic Regression achieved excellent predictive performance using standard regularization (C = 1), suggesting that the underlying relationships were adequately captured without highly complex model architectures.

Supplementary Table S2. Multicollinearity Assessment Using Variance Inflation Factors (VIF).

**Variable Removed**	**Reason**
Waist Circumference	High VIF
Diastolic Blood Pressure	High VIF
Male Partner Age	High VIF
Amenorrhea	High correlation
Progressively Motile Sperm Cells	High VIF
Total Sperm Count	High VIF
Pain in Intercourse	Near-zero variance
Fibroid	Near-zero variance

Supplementary Table S3. Variables Removed During Sensitivity Analysis.

Variable	VIF
Systolic Blood Pressure	513.33
Diastolic Blood Pressure	390.98
Waist Circumference	201.99
First Period Age	116.40
BMI	113.12
Patient Age	102.28
Male Partner Age	97.99
Fasting Glucose	82.98
High-Density Lipoprotein Cholesterol (HDL-C)	42.20
Total Motile Sperm Cells	31.20
Progressively Motile Sperm Cells	22.34
